# Analyzing Mortality Risk and Medical Burden among Patients with Traumatic Brain Injury and Subsequent Dementia

**DOI:** 10.3390/jcm8050686

**Published:** 2019-05-15

**Authors:** Dorji Harnod, Tomor Harnod, Cheng-Li Lin, Chia-Hung Kao

**Affiliations:** 1 Department of Emergency and Critical Care Medicine, Fu Jen Catholic University Hospital, New Taipei City 243, Taiwan; A00635@mail.fjuh.fju.edu.tw; 2 School of Medicine, College of Medicine, Fu Jen Catholic University, New Taipei City 243, Taiwan; 3 Department of Neurosurgery, Hualien Tzu Chi General Hospital, Buddhist Tzu Chi Medical Foundation, Hualien 970, Taiwan; 4 College of Medicine, Tzu Chi University, Hualien 970, Taiwan; 5 Management Office for Health Data, China Medical University Hospital, Taichung 404, Taiwan; a21467@mail.cmuh.org.tw; 6 College of Medicine, China Medical University, Taichung 404, Taiwan; 7 Graduate Institute of Biomedical Sciences, College of Medicine, China Medical University, Taichung 404, Taiwan; 8 Department of Nuclear Medicine and PET Center, China Medical University Hospital, Taichung 404, Taiwan; 9 Department of Bioinformatics and Medical Engineering, Asia University, Taichung 413, Taiwan

**Keywords:** cohort study, mortality, national health insurance, posttraumatic dementia, traumatic brain injury

## Abstract

We used the National Health Insurance Research Database of Taiwan to determine whether patients with posttraumatic dementia (PTD) exhibit increased mortality and medical burden than those without it. Patients ≥20 years of age having head injury admission (per the International Classification of Diseases, Ninth Revision, Clinical Modification (ICD-9-CM) codes 850–854, 959.01) between 2000 and 2012 were enrolled as traumatic brain injury (TBI) cohort. A PTD cohort (with ICD-9-CM codes 290, 294.1, 331.0) and a posttraumatic nondementia (PTN) cohort were established and compared in terms of age, sex, and comorbidities. We calculated adjusted hazard ratios (aHRs) and 95% confidence intervals (CIs) of all-cause mortality risk, number of hospital days, and frequency of medical visits in these cohorts. Patients with PTD had a higher mortality rate than did patients with TBI alone (rate per 1000 person-years: 12.00 vs. 6.32), with an aHR of 1.54 (95% CI: 1.32–1.80). Patients with PTD who were aged ≥65 years (aHR = 1.54, 95% CI: 1.31–1.80) or male (aHR = 1.78, 95% CI: 1.45–2.18) exhibited greatly increased risks of mortality. Furthermore, patients with PTD had 19.9 more hospital days and required medical visits 4.49 times more frequently compared with the PTN cohort. Taiwanese patients with PTD had increased mortality risk and medical burden compared with patients who had TBI only. Our findings provide crucial information for clinicians and the government to improve TBI and PTD outcomes.

## 1. Introduction

Traumatic brain injury (TBI) is common around the world, and it is associated with substantial early and long-term mortality, disabilities, costs, and subsequent development of various neurological deficits [1,2]. Patients surviving the early stage of TBI are usually at higher risk of developing disabilities and comorbidities in their later lives. TBI was reported to decrease victims’ life expectancy by six years, regardless of their age at the time of the accident [3]. Baguley et al. reported that patients experiencing TBI had 5 to 10 times higher risks of mortality from various causes, such as respiratory illnesses, newly developed neurologic disorders, mental disorders, or digestive diseases [4].

Dementia is a neurological degenerative disorder, and its prevalence is gradually increasing as populations are aging. The prevalence of dementia is expected to double over the next 20 years because of a prolonged average lifespan and progressive changes of population demographics [5,6]. Some common risk factors are known for the pathogenesis between dementia and various disorders, and these lead to victims’ mortality. Several studies have suggested that patients affected by various forms of TBI, such as battle injury and sports injury, may develop posttraumatic dementia (PTD) [7,8]. A retrospective study in Taiwan revealed that 2.66% of patients with TBI developed PTD during a five-year follow-up period [9]. However, similar studies have found no relationship between dementia and brain injury [10,11,12]. Current inconsistencies in the association of TBI with subsequent dementia prompt further questions regarding whether PTD affects the survival outcomes of patients suffering from TBI. More solid study data are required that establish the causal relationships among TBI, PTD, and patients’ survival outcomes. 

Therefore, we used a nationwide, population-based database in Taiwan to analyze and compare the mortality risks and risk factors of patients with PTD and those with TBI but without PTD. Because Taiwan is representative of east Asian societies and Taiwanese people are similar to people in China and Southeast Asia [13], the findings of this study may aid future development and implementation of effective treatment strategies in other Asian countries. 

## 2. Methods

### 2.1. Data Source

Taiwan’s National Health Insurance (NHI) program established the National Health Insurance Research Database (NHIRD) in 1995, and this contains health claims data of nearly 99% of Taiwanese citizens. The database possesses several advantages [14]. The first is that the database was reliably compiled by the government. Second, the individuals from the database were followed up long term. Finally, the database includes complete information such as that regarding inpatient, outpatient, and other individual medical services. Moreover, concerning individuals’ privacy, the identification numbers were encrypted before the database was released. In this study, we used the inpatient files in database available for 1996 to 2013. The diagnoses for the NHI were coded according to the International Classification of Diseases, Ninth Revision, Clinical Modification (ICD-9-CM) during the study period. The Research Ethics Committee of China Medical University and Hospital in Taiwan approved the study (CMUH104-REC2-115-CR3). 

### 2.2. Data Availability Statement

The dataset used in this study is held by the Taiwan Ministry of Health and Welfare (MOHW) (https://www.mohw.gov.tw/mp-2.html). The Ministry of Health and Welfare must approve our application to access this data. Any researcher interested in accessing this dataset can submit an application form to the Ministry of Health and Welfare requesting access. For further assistance, please contact the staff of MOHW (Email: stcarolwu@mohw.gov.tw). Taiwan Ministry of Health and Welfare Address: No.488, Sec. 6, Zhongxiao E. Rd., Nangang Dist., Taipei City 115, Taiwan (R.O.C.). Phone: +886-2-8590-6848. All relevant data are within the paper.

### 2.3. Study Population

In this study, we defined two cohorts: a PTD cohort and a posttraumatic nondementia (PTN) cohort. According to the guideline of NHI in Taiwan, a patient with mild head injury should be treated at outpatient service if the patient’s consciousness is clear, and do not show any intracranial hemorrhage or brain contusion by brain image. For the patients admitted, head injuries had to qualify as moderate or severe head injuries with TBI from 1 January 1996 to 31 December 2012. The PTD cohort was comprised patients aged ≥20 years who were admitted with a head injury diagnosis (ICD-9-CM 850–854, 959.01) and who received a subsequent diagnosis of dementia (ICD-9-CM 290, 294.1, 331.0) after the TBI admission from 1 January 2000 to 31 December 2012. The dementia diagnosis date was set as the index date. The date of enrollment for the PTN cohort was matched with the same year of the index date of the subjects with PTD while the month and the day were randomly assigned. The PTN cohort comprised patients admitted with TBI who had no diagnosis of dementia after the trauma and who had frequency matched approximately 1:1 with the PTD cohort for age (in a 5-year span), sex, and index year. The exclusion criteria eliminated patients less than 20 years old, who had received a dementia diagnosis before their TBI, or who died during the TBI admission due to a direct cause from the severe trauma itself. Each of the study subjects was followed until death, until the patients were censored for withdrawal from the database, or the end of 2013, whichever came first. We investigated comorbidities presented before the index date, namely anxiety (ICD-9-CM 300), mental disorders (ICD-9-CM 290–319), diabetes mellitus (ICD-9-CM 250), hypertension (ICD-9-CM 401–405), hyperlipidemia (ICD-9-CM 272), cerebrovascular disease (ICD-9-CM 430–438), chronic kidney disease (ICD-9-CM 580–589), and Charlson-comorbidity index (CCI). The CCI is a scoring system that includes weighting factors on important concomitant diseases; it has been validated for use with the ICD-9-CM coded administrative database [15,16]. We categorized the CCI into 4 levels: 0, 1, 2, and 3 or more. The CCI was counted for each subject from claim data of outpatient visits or hospitalizations before the index date. 

### 2.4. Statistical Analysis

The demographic factors and comorbidities were determined for the two cohorts. We used *t* testing for continuous variables and chi-square testing for category variables to compare differences between the cohorts from the index date until death, withdrawal from the database, or December 31, 2013. To estimate the risk of the mortality for the PTD and PTN cohorts, hazard ratios (HRs), adjusted HRs (aHRs), and 95% confidence intervals (CIs) were calculated using univariable and multivariable Cox proportional hazard models, respectively. The mortality rates in the two cohorts were measured, and the cumulative incidences of the PTD and PTN cohorts were compared through the Kaplan-Meier method and determined by a logrank test. We used multiple stepwise linear regression analyses to identify independent risk factors associated with hospital days per year and frequency of medical visits per year. The stepwise selection process consisted of series of alternating forward selection and backward elimination steps. The variable had to be significant at the 0.05 level before it was entered into the model, and a variable in the model had to be significant at the 0.05 level for remaining in the model. All statistical analyses were performed using SAS statistical software, version 9.4 (SAS Institute Inc., Cary, NC, USA). The cumulative incidence curve was plotted by R software. The significance criteria used a 2-tailed *p* value < 0.05. 

## 3. Results

We enrolled 3448 patients in total, with 1725 in the PTD cohort and 1723 in the PTN cohort. Among the patients, 57.6% were male, and approximately 82% were more than 65 years of age. The mean ages of the PTD and PTN cohorts were 74.6 and 73.6 years, respectively. Matching by age, sex, and index year meant that no significant differences existed between the two cohorts in terms of grouped age (*p* = 0.98) or sex (*p =* 0.99). Comorbidities, except hyperlipidemia (*p =* 0.23), were significantly more prevalent in the PTD cohort. Patients of the PTD cohort were more prevalent with CCI than in the PTN cohort (Table 1).

**Figure 1:**
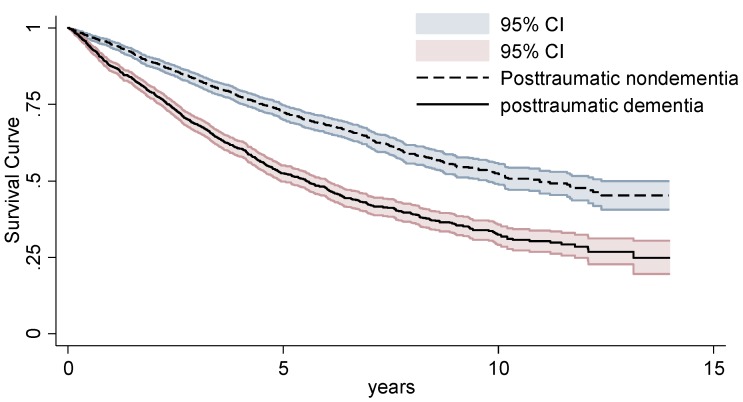
Survival curve between posttraumatic patients with and without posttraumatic dementia.

As seen in Figure 1, the 14-year cumulative incidence of mortality estimated through the Kaplan-Meier method revealed that mortality rate was considerably higher in the PTD cohort than in the PTN cohort (*p <* 0.001) (Figure 1). 

Comparison of the overall mortality rates and HRs of the two cohorts revealed that the mean (range) follow-up times were 4.10 (0.03–14.0) and 5.13 (0.03–14.0) years, and the mortality rates (per 1000 person-years) were 12.00 and 6.32 in the PTD and PTN cohorts, respectively. Table 2 reveals that when we categorized and stratified patients by age, sex, and comorbidities for analysis. The PTD cohort had 1.89 times greater mortality risk (HR = 1.89, 95% CI: 1.70–2.10) compared with the PTN cohort. After adjustment for age, sex, and all comorbidities, a 1.54-fold increased risk of mortality remained (aHR = 1.54, 95% CI: 1.32–1.80) in the PTD cohort compared with the PTN cohort. Patients with PTD who were aged 65 years or more (aHR = 1.54, 95% CI: 1.31–1.80), male (aHR = 1.78, 95% CI: 1.45–2.18), or without comorbidity (aHR = 8.08, 95% CI: 3.33–19.6) had greatly increased risks of mortality compared with the PTN cohort (Table 2).

The aHR of mortality was increased 1.06-fold (95% CI = 1.06–1.07) with age (every year) and increased 1.39-fold for men relative to women (95% CI = 1.25–1.55). The risk of mortality was increased in patients with comorbidities, namely diabetes mellitus (aHR = 1.37, 95% CI = 1.20–1.57), and chronic kidney disease (aHR = 1.22, 95% CI = 1.07–1.39). Compared to patients with CCI score 0, patients with CCI score 1 (aHR = 1.38, 95% CI = 1.15–1.67), patients with CCI score 2 (aHR = 1.61, 95% CI = 1.31–1.98), and patients with CCI score ≥ 3 (aHR = 2.43, 95% CI = 1.96–3.01) had higher mortality risks. 

For patients with PTD versus PTN cohorts with subgroups of CCI score 1, CCI score 2, and CCI score ≥ 3, the aHRs of mortality were 1.58, 1.54, and 1.60, respectively. Unlike the patients with PTD without comorbidity had a very higher mortality risk (aHR = 8.08), patients with PTD who had one or more comorbidity exhibited little increased mortality risk (aHR = 1.26, 95% CI: 1.23–2.08). In the patients with <2 years of follow-up, the PTD cohort had a higher increased mortality risk compared with the PTN cohort (aHR = 1.71, 95% CI = 1.32–2.20). However, the mortality risks in all PTD subgroups with different follow-up time had significantly higher mortality risks than that in PTN cohort (Table 2). 

Risk factors associated with the increased frequency of medical visits per year appear in Table 3. PTD, male sex, and comorbidity of depression, anxiety, mental disorders, diabetes mellitus, hypertension, hyperlipidemia, and chronic kidney disease were strongly associated with the increased frequency of medical visits per year in the study cohorts. Patients with PTD required 4.49 times more medical visits per year compared with the PTN cohort (Table 3). We further analyzed the risk factors for increased all-cause admission in posttraumatic patients. The hospital days would increase 0.36 days per year of additional age. PTD, male sex, and comorbidities of stroke, chronic kidney disease, and diabetes mellitus were the major risk factors for increased number of hospital days. Patients with PTD had a 19.9 more hospital days per year compared with the PTN cohort (Table 4). 

## 4. Discussion 

Taiwanese patients with both TBI and PTD had a higher mortality risk than those with TBI and without PTD. Overall, posttraumatic patients with and without PTD had mortality rates of 12.00 and 6.32 per 1000 person-years, respectively. The known mortality rate for the general population in 2013 was 435.3 per 100,000 according to the Taiwan NHI database [17]; therefore, the mortality risk in our study’s patients with PTD was higher than this mark. We enrolled both patients with first-time TBI and those with a history of multiple TBI to estimate any possible correlations with long-term survival outcome and PTD. The large study population, being fairly unselected and excluding patients with only mild head injury treated through outpatient services, constituted the main strength of this study. We noted that PTD development can be a specific predictor for poor long-term survival outcomes after TBI in Taiwan, irrespective of existing comorbidities. Moreover, PTD greatly increases the number of hospital days and yearly medical visits for posttraumatic long-term care. All of this evidence suggests that PTD may directly increase the burden of patient care on families, societies, and the government. 

Our findings from the 14-year follow-up cohort study of PTD and survival outcomes could be considered as furthering the research of several other population-based studies. Gardner et al. found a 1.26-fold increased risk of dementia development over seven years of follow-up in patients who had a TBI diagnosis and who were aged more than 55 years [18]. Barnes et al. reported that moderate to severe TBI potentially increased PTD risk across all ages whereas mild TBI increased it in those older than 65 years [19]. In another cohort study using the NHIRD, 2.66% of patients with TBI developed dementia during a five-year follow-up period and had a 1.68-fold increased risk of dementia after adjustments for sociodemographic factors and several comorbidities [9]. Our study results further suggest that patients with PTD exhibit greater increases in long-term mortality risks and medical burdens among posttraumatic patients than previously believed. In addition, male patients and patients ≥65 years of age are exposed to a much higher risk of mortality. After adjustments for other variables, male sex and comorbidities of depression, anxiety, mental disorders, diabetes, hypertension, hyperlipidemia, and chronic kidney disease represented further increases to the posttraumatic inpatient burden. The relationships among increased numbers of hospital days, more frequent medical visits, certain comorbid disorders, and PTD-related mortality interact to increase the subsequent medical burden associated with these patients. Because dementia cannot currently be effectively prevented, understanding the risk factors and outcomes of TBI and PTD is a practical approach to develop future strategies for reducing posttraumatic mortality risk in daily clinical services. The data in this study imply that patients with TBI and subject to certain factors require additional preventative treatments. 

Posttraumatic patients who develop PTD are less likely to return to their ordinary lives than are those who not develop PTD. The mechanisms of PTD development are complex and unclearly understood. Several reasons could be proposed for the increased risk of PTD among patients with TBI who are usually diagnosed with unspecific dementia. First, TBI may induce a chronic inflammatory process, perhaps over a lifelong period. Studies have demonstrated that TBI is associated with increased levels of cytokines and microglial activation [20,21,22]. If long-term inflammatory immune alterations occur in the brain, persistent damage may result in diffuse brain dysfunction. Second, the evidence has suggested that tau and amyloid-β deposition may be noted after TBI and resemble the deposition process seen in Alzheimer’s dementia [23]. Third, patients with TBI might have had sedentary lifestyles with poor sleep, depression, or even alcohol abuse. The potential of such lifestyles to increase the risk of dementia was addressed [24]. 

In this study, deaths during TBI-related admission were excluded because these deaths might have been directly caused by the severe trauma itself. All analyses were based on inpatient data for high validity in this study. However, this study had several limitations. First, we could not directly contact the patients because their identities were anonymized in the longitudinal health insurance database (LHID). Therefore, we could not analyze details regarding the severity of their TBI or the psychological burden of TBI and PTD to control the confounding effects from actual medications used. Second, our data set included the all-cause mortality of posttraumatic patients who died at inpatient facilities only. Rarely, individual deaths might have occurred outside the hospital and thus outside the scope of our study. Third, although the NHI program covers nearly 99% of Taiwan’s citizens and guarantees equality of access to inpatient services and of outcomes for everyone at hospitals throughout the country [14,25], rare miscoding may have nevertheless occurred in the LHID. However, this study presents statistically significant results to demonstrate the increased subsequent mortality risk in patients with PTD. 

## 5. Conclusions

Taiwanese patients with TBI and PTD had a higher mortality risk than those with TBI and without PTD; the highest risk was noted in male patients aged 65 years or more. Moreover, hospital days and frequency of medical visits increased in patients with PTD. Additional studies in other countries are required to confirm whether this result is globally applicable.

## Figures and Tables

**Table 1:** Distribution of age, sex, and comorbidity in the posttraumatic dementia and posttraumatic nondementia cohorts.

	Posttraumatic Nondementia *N* = 1723		Posttraumatic Dementia *N* = 1725		
	*n*	%	*n*	%	*p*-Value
Age, year					0.98
≤64	306	17.8	306	17.7	
≥65	1417	82.2	1419	82.3	
Mean (SD) ^§^	73.6	12.8	74.6	12.9	0.03
Sex					0.99
Female	731	42.4	732	42.4	
Male	992	57.6	993	57.6	
Comorbidity					
Depression	106	6.15	331	19.2	<0.001
Alcohol-related illness	106	6.15	145	8.41	0.01
Anxiety	444	25.8	684	39.7	<0.001
Mental disorders	786	45.6	1654	95.9	<0.001
Diabetes mellitus	274	15.9	418	24.2	<0.001
Hypertension	1143	66.3	1370	79.4	<0.001
Hyperlipidemia	582	33.8	616	35.7	0.23
Cerebrovascular disease	257	14.9	605	35.1	<0.001
Chronic kidney disease	294	17.1	406	23.5	<0.001
CCI score					<0.001
0	1016	59.0	0	0.00	
1	353	20.5	541	31.4	
2	173	10.0	519	30.1	
3 or more	181	10.5	665	38.6	

Chi-squared test for categorical variables and ^§^ t-test for continuous variables. CCI score = Charlson comorbidity index score.

**Table 2:** Incidence and hazard ratios of mortality stratified by age, sex, and comorbidity comparing patients with posttraumatic dementia and patients with posttraumatic nondementia.

Variable	Posttraumatic Nondementia		Posttraumatic Dementia		Crude HR (95% CI)	Adjusted HR ^b^ (95% CI)
	*N* = 1723		*N* = 1725			
	Deaths	Mortality Rate ^a^	Deaths	Mortality Rate ^a^		
All	559	6.32	851	12.0	1.89 (1.70, 2.10) ***	1.54 (1.32, 1.80) ***
Age, year						
≤64	35	1.84	69	3.95	2.15 (1.43, 3.23) ***	0.81 (0.43, 1.53)
≥65	524	7.55	782	14.7	1.95 (1.74, 2.18) ***	1.54 (1.31, 1.80) ***
Sex						
Female	216	5.71	312	9.72	1.69 (1.42, 2.01) ***	1.21 (0.96, 1.52)
Male	343	6.77	539	14.0	2.05 (1.79, 2.35) ***	1.78 (1.45, 2.18) ***
Comorbidity						
None	74	3.88	8	25.8	6.66 (3.20, 13.9) ***	8.08 (3.33, 19.6) ***
With any one	485	6.99	843	12.0	1.71 (1.53, 1.91) ***	1.26 (1.11, 1.43) ***
CCI score						
0	251	4.40				
1	126	7.28	190	7.70	1.05 (0.84, 1.32)	1.58 (1.15, 2.17) **
2	74	10.3	249	10.9	1.05 (0.81, 1.36)	1.54 (1.11, 2.15) *
3 or more	108	15.7	412	17.8	1.13 (0.91, 1.39)	1.60 (1.23, 2.08) ***
Follow time, years						
<2	190	5.99	357	12.0	2.01 (1.68, 2.39) ***	1.71 (1.32, 2.20) ***
2–5	208	5.31	341	10.0	1.89 (1.59, 2.25) ***	1.38 (1.08, 1.77) **
>5	161	6.34	153	9.60	1.51 (1.21, 1.88) ***	1.45 (1.03, 2.03) *

^a^ Mortality Rate, per 1000 person-years; HR, hazard ratio; CCI score = Charlson comorbidity index score; ^b^ Variables found to be statistically significant in the univariable model were further included in the multivariable model. Adjusted for age, sex, mental disorders, diabetes mellitus, hypertension, hyperlipidemia, cerebrovascular disease, chronic kidney disease, and CCI score. * *p* < 0.05; ** *p* < 0.001; *** *p* < 0.001.

**Table 3:** Stepwise regression analysis for frequency of medical visits per year.

Variable	Parameter Estimate	Standard Error	95% CI
Intercept	15.6	1.08	(13.4, 17.7) ***
Posttraumatic dementia vs. Posttraumatic nondementia	4.49	0.98	(2.57, 6.40) ***
Sex (male vs. female)	3.48	0.82	(1.87, 5.08) ***
Depression	5.72	1.33	(3.11, 8.33) ***
Anxiety	4.79	1.04	(2.74, 6.83) ***
Mental disorders	7.67	1.17	(5.37, 9.97) ***
Diabetes mellitus	4.22	1.04	(2.17, 6.26) ***
Hypertension	7.68	0.96	(5.80, 9.56) ***
Hyperlipidemia	5.23	0.90	(3.46, 6.99) ***
Chronic kidney disease	7.74	1.02	(5.74, 9.74) ***

*** *p* < 0.001.

**Table 4:** Stepwise regression analysis for hospital days per year (all-cause admissions).

Variable	Parameter Estimate	Standard Error	95% CI
Intercept	−23.3	4.92	(–33.0, −13.7) ***
Posttraumatic dementia vs. Posttraumatic nondementia	19.9	1.65	(16.6, 23.1) ***
Age (every one year)	0.36	0.06	(0.23, 0.48) ***
Sex (male vs. female)	10.7	1.63	(7.53, 13.9) ***
Stroke	9.25	1.93	(5.47, 13.0) ***
Chronic kidney disease	10.2	2.04	(6.16, 14.2) ***
Hyperlipidemia	−6.69	1.76	(0.20, 8.43) *
Diabetes mellitus	4.31	2.10	(0.20, 8.43) *

* *p* < 0.05, *** *p* < 0.001.
